# Sex Differences in the Burden and Complications of Diabetes

**DOI:** 10.1007/s11892-018-1005-5

**Published:** 2018-04-18

**Authors:** Sanne A. E. Peters, Mark Woodward

**Affiliations:** 1 The George Institute for Global Health, University of Oxford, Le Gros Clark Building, South Parks Road, Oxford, OX1 3QX UK; 2 The George Institute for Global Health, University of New South Wales, Sydney, Australia; 3 Department of Epidemiology, Johns Hopkins University, Baltimore, MD USA

**Keywords:** Diabetes, Cardiovascular disease, Men, Women, Sex differences

## Abstract

**Purpose of the Review:**

To review the latest evidence on sex differences in the burden and complications of diabetes and discuss the potential explanations for the sex differences described.

**Recent Findings:**

Diabetes is a strong risk factor for vascular disease, with compelling evidence that the relative risks of vascular diseases conferred by diabetes are considerably greater in women than men. The mechanisms underpinning women’s excess relative risk of vascular disease from diabetes are unknown. Sex differences in the health care provided for the prevention, management, and treatment of diabetes and its complications could contribute to women’s greater excess relative risks of diabetes complications. However, since the excess risk of vascular disease is not seen for other major vascular risk factors, inherent biological factors may be more likely to be involved. In addition to other cardiometabolic pathways, the sex dimorphism in body composition and fat distribution may be particularly important in explaining women’s greater excess risk of the vascular complications of diabetes.

**Summary:**

There is strong evidence to suggest that diabetes is a stronger risk factor for vascular disease in women than men. Although several mechanisms may be involved, further research is needed to provide new and deeper insights into the mechanisms underpinning sex differences in the association between diabetes and vascular diseases. Such research will inform patients, health care professionals, and policy makers to ensure that women are not disproportionately affected by diabetes, and will help to reduce the burden in both sexes.

## Introduction

Diabetes is a global epidemic and a major cause of cardiovascular disease (CVD), chronic kidney disease, blindness, and amputation. In 2017, 425 million people had diabetes and this figure is expected to continue to increase rapidly across most countries and all income levels, to an estimated 629 million globally in 2045 [1]. Diabetes also poses a substantial economic burden on individuals, communities, health care systems, and countries. [1] Halting the rise of diabetes at its 2010 levels is one of the global targets set for 2025 at the UN high-level meeting on non-communicable diseases [2].

There is increasing evidence of clinically meaningful sex differences in the aetiology, epidemiology, prevention, management, and prognosis of many, mainly non-communicable, diseases (NCDs), including diabetes. Many health organisations, funders, and publishers have called for the inclusion of a sex and gender dimension at all stages of biomedical research, as a means to safeguard and improve the quality and societal relevance of scientific research [3–5]. In recognition of the unique aspects of diabetes in women, which differ across the lifespan and societies, the International Diabetes Federation’s World Diabetes Day 2017 focussed on women and diabetes [6]. In this article, we review the current evidence on sex differences in the burden and complications, and discuss the potential explanations for the sex differences described.

## Global Burden of Diabetes

**Fig. 1:**
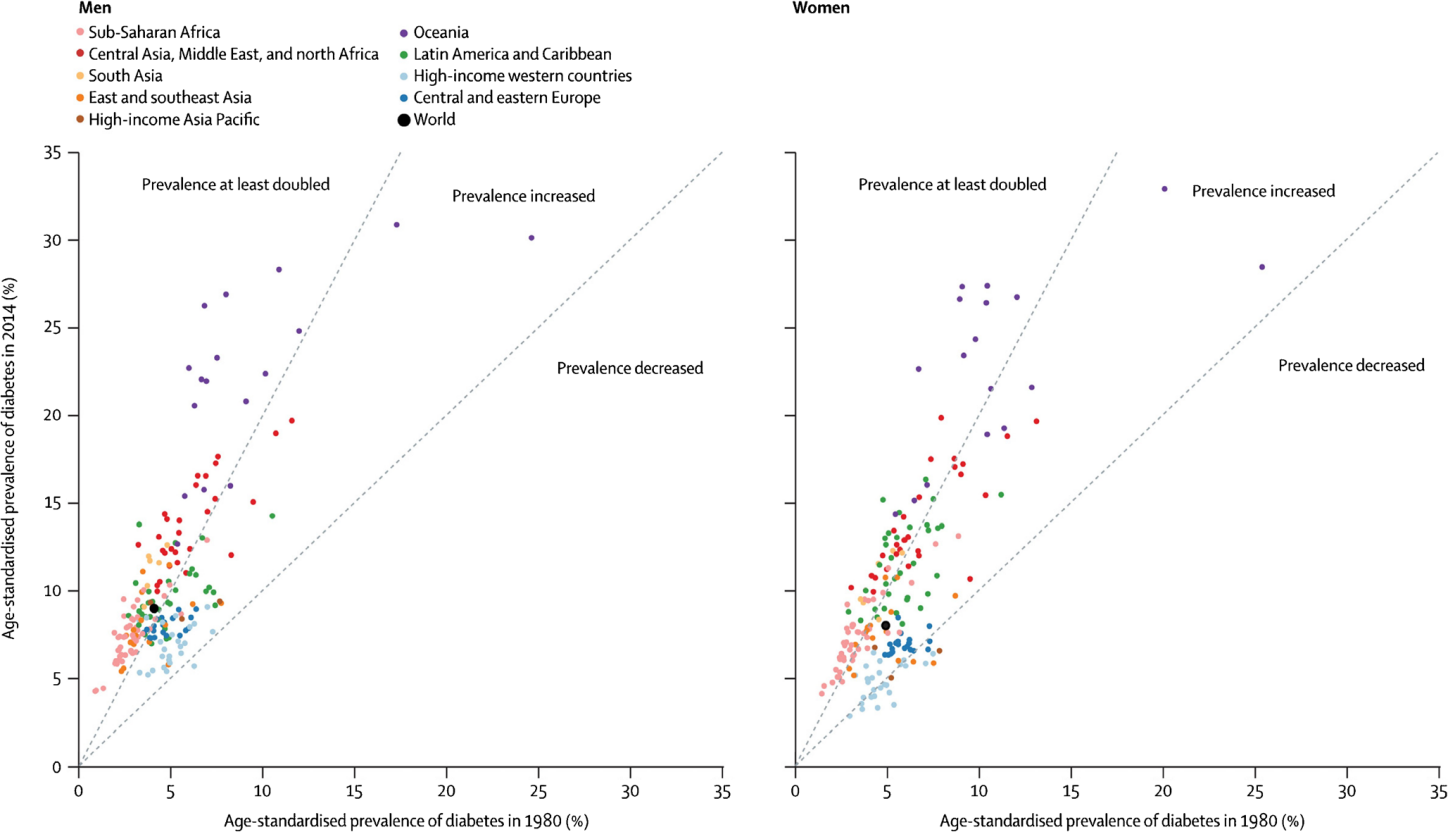
Comparison of age-standardised prevalence of diabetes in men and women in 1980 and 2014. (Reproduced from: Lancet 2016; 387(10027): 1513–30; 10.1016/S0140-6736(16)00618-8; Creative Commons user licence https://creativecommons.org/licenses/by/4.0/) [7••]

The NCD Risk Factor Collaboration (NCD-RisC) provides the most comprehensive estimates to date of the worldwide trends in the burden of diabetes [7••]. By 2014, NCD-RisC held data from 751 population-based studies with 4.4 million adults from 146 countries. All studies had collected data on diabetes through direct measurement of its biomarkers. Diabetes was defined as fasting plasma glucose of 7.0 mmol/L or higher, a history of diabetes, or the use of insulin or oral antidiabetic drugs. From these data, the global age-standardised prevalence of diabetes was estimated to have increased from about 4% in 1980 to 9% in 2014 in men and from 5% to almost 8% in women. However, these global estimates mask substantial differences in the prevalence of diabetes across regions (Fig. 1). Similarly, changes over time in the burden of diabetes differ significantly between regions, with greater rates of increase in low-income and middle-income countries than in high-income countries. In 2014, the age-standardised prevalence of diabetes in women was lowest in Western Europe, where the prevalence was below 5%. These rates were similar to those in 1980. In men, the lowest age-standardised prevalence of diabetes was 6%, in Northwestern Europe. The same region showed the least rise in the prevalence of diabetes since 1980. In contrast, men and women in Polynesia and Micronesia had the highest age-standardised prevalence (over 20%), with a 15% rise in both sexes since 1980.

Given these high levels of prevalence and increasing trends in some countries, the chance that the global UN target—of halting the rise of diabetes at its 2010 levels—will be met in 2025 was estimated to be 1% in women and lower than 1% in men [7••]. Only a few countries, mostly in Western Europe, had a chance of 50% or higher of meeting the 2025 target. On the contrary, if the post-2000 trends continue, the age-standardised prevalence of diabetes in 2025 will rise to over 10% in women and nearly 13% in men, thus increasing the global health and economic impact of diabetes even further.

## Complications of Diabetes

**Fig. 2:**
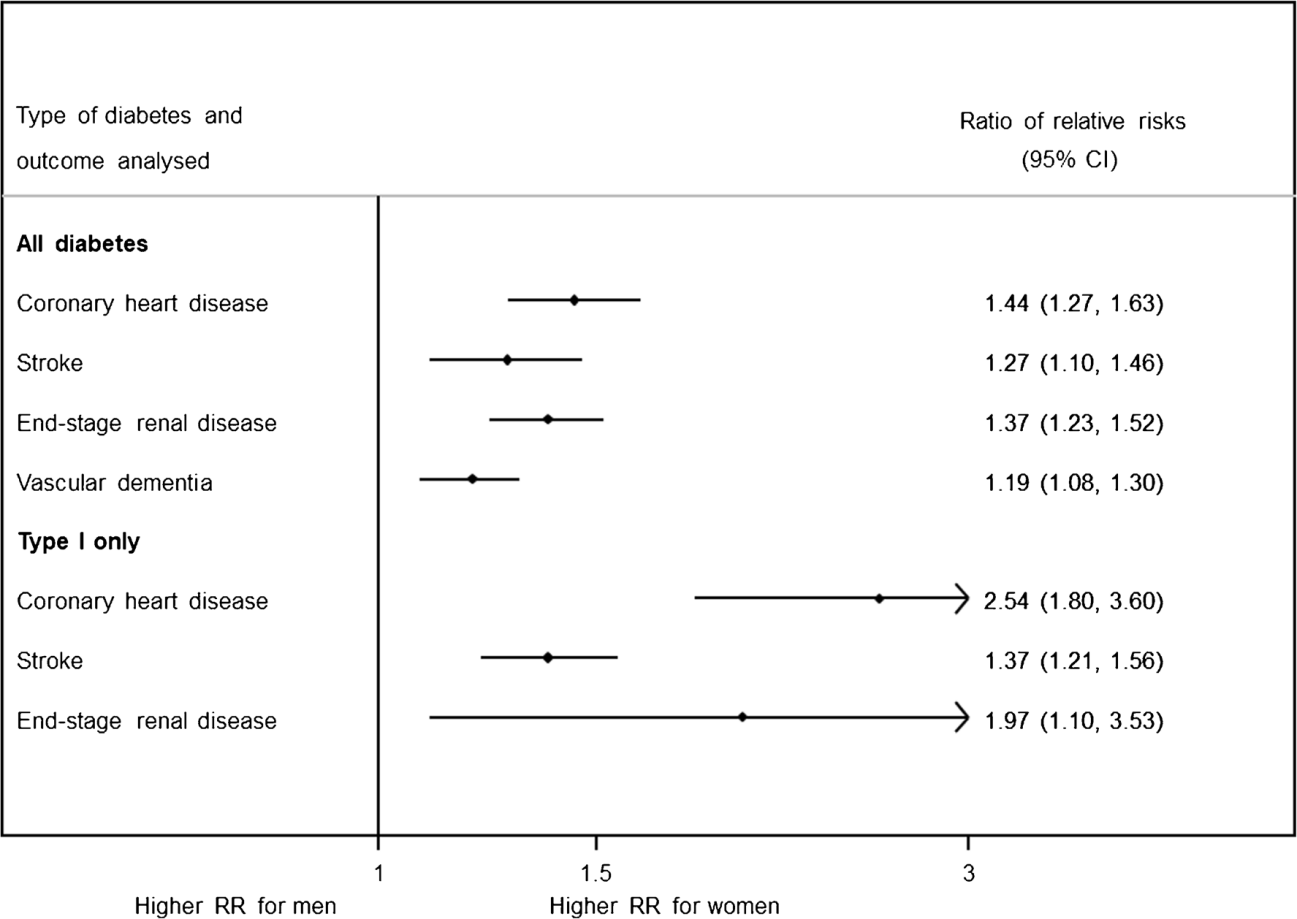
Results from prior meta-analyses of sex differences in the effects of diabetes on vascular outcomes, summarised through the ratios of women-to-men adjusted relative risks (and 95% confidence intervals) pooled across cohort studies

Diabetes is associated with an increased risk of several vascular conditions, dementia, certain cancers, respiratory disease, and infectious diseases. CVD is the most common adverse outcome of diabetes and so, unsurprisingly, more is known about sex differences in the effects of diabetes on CVD than other diseases. On average, people with diabetes have about twice the risk of CVD compared to those without diabetes [8]. However, there are differences in the relative risk (RR) of various CVD subtypes, with strong positive associations between type 2 diabetes and peripheral arterial disease, ischaemic stroke, stable angina, heart failure, and myocardial infarction, but potentially inversely associations with abdominal aortic aneurysm and subarachnoid haemorrhage [9]. Moreover, not everyone with diabetes has the same degree of excess risk of vascular disease. Large-scale meta-analyses, summarising all the evidence available to date from the best quality epidemiological studies globally, have provided compelling evidence that diabetes confers a 44% greater excess risk of coronary heart disease (CHD) and a 27% greater excess risk of stroke in women than in men, independent of sex differences in other major risk factors [10,11]. The pooled RR of CHD associated with diabetes was 2.82 (95% CI 2.35, 3.38) in women and 2.16 (1.82, 2.56) in men [11]. For stroke, the corresponding RRs were 2.28 (1.93, 2.69) in women and 1.83 (1.60, 2.08) in men [10]. Most cases included in these meta-analyses had type 2 diabetes, since this accounts for about 90% of all diabetes cases. However, a meta-analysis that focused specifically on type 1 diabetes also found that diabetes type 1 was a much stronger risk factor for premature death among women than men, which was primarily driven by sex differences in RRs of vascular events in individuals with type 1 diabetes [12]. Moreover, our recent meta-analysis reported that diabetes is associated with a 19% greater relative risk of vascular dementia among women than men with diabetes [13], whilst another group has used similar methods to show a similar sex differential for end stage renal disease [14]. Hence, sex differences in the vascular consequences of diabetes occur beyond CHD and stroke, the major components of CVD. Figure 2 summarises the meta-analyses mentioned in this paragraph, through the ratio of relative risks, women-to-men.

Women’s greater excess risk of vascular disease associated with diabetes might be challenged on the basis that women generally have lower CVD risk than men, and thus the same absolute difference must inevitably lead to higher relative risks in women [15]. This explanation is strengthened by findings that the female disadvantage lessens with age, that is, as the overall risk of CVD increases with age the women-to-men relative risk declines. However, examination of data on background risks from cohort studies does not support the idea that the sex differences reported above are a mathematical artefact. Moreover, our analyses of other CVD risk factors have not always shown female relative risks to be higher than men. Indeed, we found no evidence for a sex differential for in the risk of CHD and stroke associated with increases in BMI and high blood pressure [16,17], and for high total cholesterol, we found some indication that men have the higher relative risk of CVD [18].

While most studies have found sex differences in the diabetes-CVD relationship, there are some notable exceptions, including a large-scale study in Mexico City [19]. However, this study also found a lack of association between increased adiposity and diabetes, which is also unusual. The authors reported that this was likely due to the high prevalence of overweight and obesity in their cohort, which is an intriguing suggestion, worthy of further research. Based on the totality of evidence, our own conclusion is that there is, indeed, a real additional vascular disadvantage from diabetes amongst women. The challenge, then, is to explain why this is the case, so as to seek new treatments or policies that will not only disproportionally benefit women, but also lead to more tailored clinical care for men.

## Management of Diabetes

One of the main goals in the management of diabetes is to prevent or delay the onset of its complications. Those with diabetes therefore require treatment and control of glucose, lipid, and blood pressure levels, in addition to maintaining or achieving healthy lifestyle targets characterised by non-smoking, sufficient physical activity, weight control, and a balanced diet. Moreover, regular screening for microvascular complications in the eyes, kidneys, and feet is recommended in clinical guidelines in most countries. Sex differences in the health care provided for the prevention, management, and treatment of diabetes and its complications may therefore contribute to women’s greater excess relative risks of diabetes complications. This would be possible if women receive poorer care following their diagnosis of diabetes than men; for instance, due to physician bias. That is, women could do worse because they are less likely to be given the recommended health care. Historically, women had poorer risk factor profiles and often received poorer cardiovascular care than men. This is despite evidence for the lack of differences between women and men in the safety and effectiveness of medications to regulate lipid and blood pressure levels [20,21]. Although, in many countries, access to, and uptake of, care has become more equitable between the sexes over the past decade, recent evidence suggests that women with diabetes are still less likely than men to receive guideline-recommended care, even in the most developed nations. For instance, our own contemporary work has found sex differences, to the detriment of women, in primary and secondary cardiovascular prevention [22,23]. This is undoubtedly a factor in the female disadvantage in diabetes. For example, amongst people with diabetes, an American study reported that women were 25% less likely to achieve target cholesterol levels than men [24], whilst women with diabetes in the UK were 15% less likely than their male counterparts to receive guideline-based care or to meet treatment targets [25]. Findings from a study in a Dutch primary care setting indicated that a substantial proportion of type 2 diabetes patients who experienced an acute myocardial infarction did not receive optimal evidence-based secondary cardiovascular prevention, with women being less likely to receive repeat prescriptions than men [26]. Thus, sex differences in the healthcare provided for the prevention, management, and treatment of diabetes and its complications could well contribute to women’s greater excess relative risks of diabetes complications, particularly in those parts of the world where access to care in women is more limited than in men. However, this under-treatment for women is not restricted to those with diabetes, and there is no reason to suppose things should be worse in relation to diabetes than to other major risk factors or comorbidities. Thus, since some other risk factors for CVD, such as high blood pressure or elevated total cholesterol [17, 18], do not exhibit a female disadvantage, physician bias cannot be the only explanation for the sex differential in diabetes.

Another possible cause of women’s additional risk from diabetes is that they are less aware of their risk of CVD, or are less likely to adhere to treatment recommendations once they are at high risk of CVD. For instance, an American study found adherence to antidiabetic medication to be slightly lower amongst women than men [27]. This leads one to consider the differential social structures contrasting the sexes. It might be that women are more concerned about their families than themselves, or just are less aware of their CVD risks as much as do men. However, this again is unlikely to be specific to diabetes.

## Biological Factors

Most likely, natural biology has a large influence on the sex differential in vascular diseases associated with diabetes. Women generally have more favourable levels of cardiovascular risk factors than men, but this pattern is not seen with deterioration in glycaemic control and progression towards diabetes [28••, 29••]. Several studies have shown that the differences in risk factor levels between diabetic and non-diabetic individuals are greater in women than in men—particularly for anthropometric variables [30, 31]. Thus, differences between women and men in the prevalence of overweight and obesity and, potentially more importantly, the sex dimorphism in body composition and fat distribution may be involved.

Overweight and obesity are key risk factors for the development of diabetes and progression to its complications. As with diabetes, halting the rise in the prevalence of obesity at its 2010 levels has been included among the UN’s global NCD targets for 2025 [2]. Global trends in adult body mass index (BMI), however, suggest that the probability of meeting the global obesity target is virtually zero [32]. Estimates from NCD-RisC demonstrate that the global age-standardised prevalence of obesity, defined as a BMI ≥ 30 kg/m^2^, has increased from 3% in 1975 to 11% in 2014 in men and from 6 to 15% in women. Two percent of the world’s men and 5% of women were severely obese (BMI ≥ 35 kg/m^2^) and 0.6% of men and 1.6% in women are morbidly obese. Hence, if these trends continue, the global prevalence of obesity is expected to increase to 18% in men and to exceed 21% in women in 2025. The prevalence of overweight and obesity differs vastly between regions, in a similar way to diabetes.

However, while the global prevalence of obesity is greater in women than in men (15 vs. 11%) [32], as indicated above the prevalence of diabetes is slightly lower in women than in men (8 vs. 9%) [7••]. These figures raise the possibility that men, on average, develop diabetes at lower levels of BMI than women. Both the Scottish diabetes registry and the UK general practice research database found that women had a mean BMI of almost 2 kg/m^2^ higher than men when first diagnosed with diabetes, despite similar levels of HbA1c [33,34]. Moreover, among 500,000 participants in the UK Biobank, mean BMI levels differed more between diabetic and non-diabetic women than between diabetic and non-diabetic men [35]. In contrast, differences in the waist-to-hip ratio between individuals with and without diabetes were broadly similar between the sexes. This difference underscores the potential role of sex differences in body composition and fat distribution in the development and progression of diabetes [36,37].

BMI is a measure of general adiposity that does not discriminate between adipose tissue present in visceral and subcutaneous areas. In contrast, measures of fat distribution, such as waist-to-hip ratio, capture both the amount of subcutaneous fat, which is relatively benign, and visceral fat, which is more metabolically adverse and closely related to insulin resistance [37]. Women tend to have more subcutaneous fat and less visceral fat than men, which is reflected in a lower waist-to-hip ratio at a given BMI. Due to a lower capacity to store fat in subcutaneous tissue in men, excess adipose tissue is more rapidly stored into visceral and ectopic tissues, including the liver, skeletal tissue, and possibly the pancreas. More rapid accumulation of fat in visceral and ectopic tissues, in turn, leads to a faster transition to insulin resistance and diabetes. Women, on the other hand, need to attain higher levels of BMI to reach the same levels of visceral and ectopic fat required to become insulin resistant and so to develop diabetes. In support of this, the greater relative increases in many cardiometabolic risk factors, including greater changes in levels of blood pressure, lipid levels, and inflammatory markers, among women with diabetes compared with men with diabetes, are in part explained by women’s greater increase in adiposity and insulin resistance, linked to diabetes [28••, 29••]. Thus, inherent sex differences in body composition and fat distribution, which may be linked to nature’s preparation for potential childbearing and lactation, might provide women with more cardiometabolic reserves, requiring a greater metabolic deterioration to develop diabetes, which may partially be responsible for women’s greater excess risk of the complications of diabetes [28••].

Further evidence for a role of body composition comes from large genome-wide association studies (GWAS), which reported a strong sex dimorphism in the genetic regulation of traits related to waist and body fat distribution, but not for height, weight, BMI, or hip circumference [38]. Furthermore, several reproductive health factors, including age at menarche, age at menopause, and childbearing history are associated with body adiposity [35,39], suggesting that reproductive factors may be involved in the development of diabetes, as well as CVD [40–42].

The diagnosis of diabetes is based on a threshold value of fasting blood glucose or glycated haemoglobin. However, hyperglycaemia is a continuous trait and there is strong evidence of a progressive association between various measures of glycaemia and the risk of macrovascular disease, both above and below the clinical threshold for diabetes [8,43]. Nevertheless, the possibility that the process of developing diabetes takes longer in women than men is supported by a study that reported that men, on average, have prediabetes for 8 years before they convert to diabetes compared with 10 years in women [44]. This prediabetic state, with elevated levels of blood glucose that are not considered high enough for a diagnosis of diabetes, could result in considerable vascular damage resulting from a prolonged state of suboptimal, untreated, glycaemic levels. While there is convincing evidence for a stronger effect of diabetes on the excess risk of vascular in women than in men, these estimates are generally not stratified by levels of glycaemia. Hence, it remains uncertain whether there are sex differences in the shape or gradient of the relationships between indices of glycaemia and vascular dysfunction and complications.

## Gestational Diabetes

Gestational diabetes is a heterogeneous condition characterised by glucose intolerance that is first detected during pregnancy [45]. Although gestational diabetes primarily affects overweight and obese women, genetic factors may also be involved [46,47]. Glucose homoeostasis restores to non-pregnancy levels shortly after delivery. Nevertheless, women with a history of gestational diabetes are at a sevenfold increased risk of developing type 2 diabetes in the future compared with those who have had a normoglycemic pregnancy [48]. Current guidelines recommend that women who have had gestational diabetes should have a glucose tolerance test after delivery [49]. Uptake of screening for type 2 diabetes is low, however, with a US study demonstrating that less than 25% of women with a diagnosis of gestational diabetes received recommended screening postnatally [50]. Clearly, the increased risk of type 2 diabetes in affected women should motivate them, and their health care practitioners, to participate in screening programmes to prevent or delay the onset of type 2 diabetes. Intriguingly, recent studies have suggested that, compared with those carrying a girl, pregnant women carrying a male fetus have a poorer β-cell function in pregnancy and a small but significant increased risk of gestational diabetes [51]. While the underlying mechanism is unclear, these findings indicate that sex differences already exist early in life and might not only impact the maternal glucose metabolism but also that of the infant.

## Non-vascular Complications

**Fig. 3:**
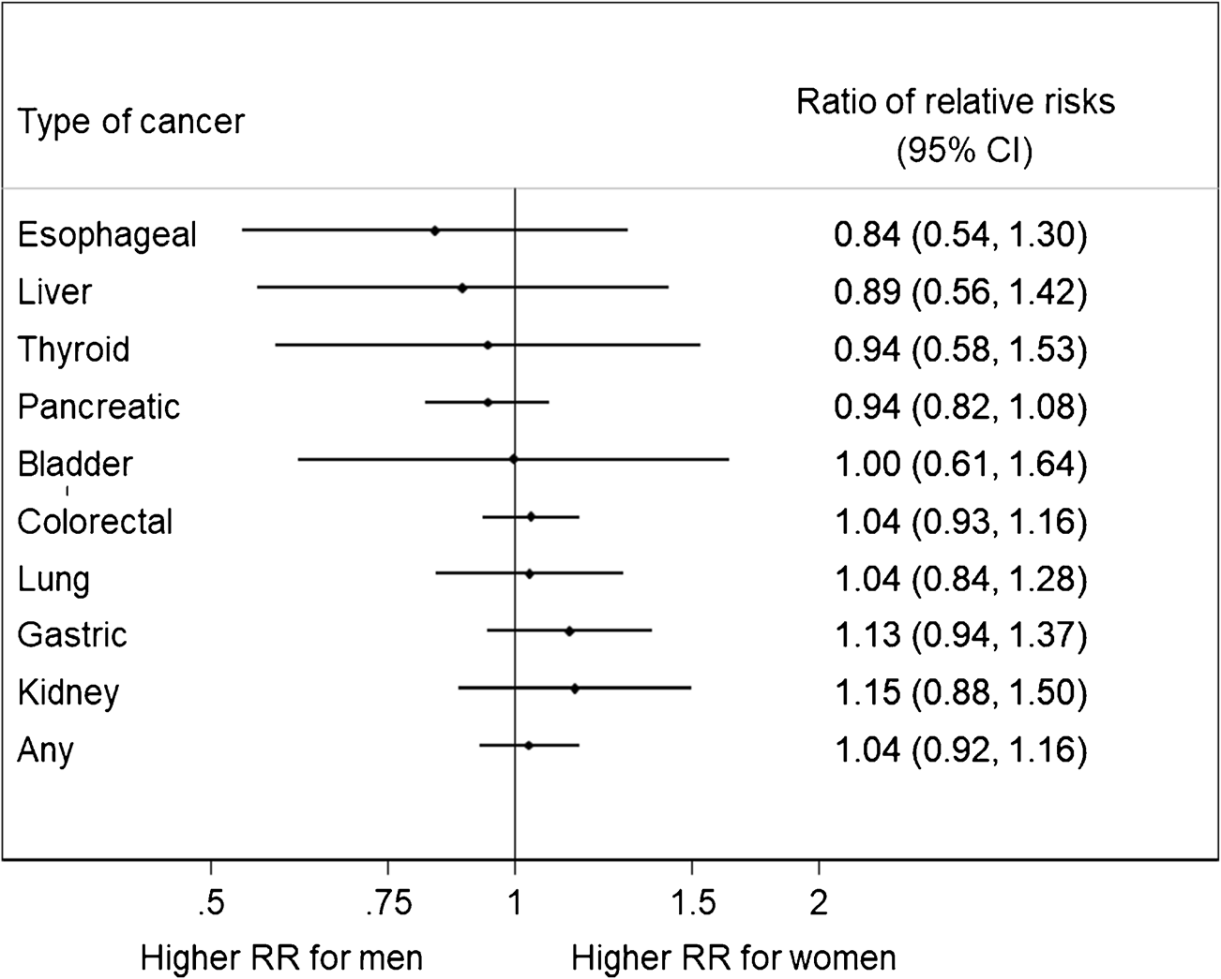
Results from prior meta-analyses of sex differences in the effects of diabetes on cancer, summarised through the ratios of women-to-men adjusted relative risks (RR) (and 95% confidence intervals) pooled across observational studies. The results for colorectal, liver, lung, and any cancer are from cohort studies only; the rest also include case-control studies

As far as we are aware, there are no compelling data showing sex differences in the relationship between diabetes and other non-vascular diseases. For instance, several meta-analyses have reported sex-specific associations between diabetes and different types of cancer (Fig. 3) [52–60]. As is common in this field, many of the studies pooled in these meta-analyses were case-control studies, which are more susceptible to bias—which could be differential by sex—than the cohort studies used in the vascular meta-analyses of Fig. 2. Furthermore, they often include studies of a single sex only, which could introduce further bias when the sexes are compared. With these caveats, from Fig. 3, one can conclude that there is no evidence to suggest that any effect diabetes has on cancer differs by sex. Moreover, we have found no evidence for a sex difference for mortality from cancer, as well as from accidents and suicide, associated with type 1 diabetes [12].

## Future Directions

There is strong evidence that the relative risks of vascular diseases conferred by diabetes are considerably greater in women than men, and no known evidence of any other sex disparities in the disease burden following diabetes. Despite the potential explanations described above, the evidence is incomplete and the reasons behind women’s excess vascular relative risk from diabetes are not fully understood. Further research is, therefore, needed to provide further insights. Specifically, sex-specific results should always be presented, not only when there is a sex-specific hypothesis, but purely as a matter of routine. Moreover, new analyses of large-scale contemporary population-based studies are needed to confirm and refine the current estimates. Such studies can overcome the inherent limitations of prior meta-analyses based on published data, which include substantial heterogeneity between studies in design and both the number and types of variables adjusted for, as well as restricted options for subgroup analyses. Given substantial differences in the risk of CVD across the lifespan, detailed subgroup analyses of sex differences across the age spectrum would be particularly valuable. Provided that extensive phenotypic and genotypic data are available, such studies will also be crucial in establishing causality of the sex differences, for example through Mendelian randomisation analyses, and in seeking biomechanical explanations. Finally, linked routinely collected electronic health record data provide a good source for identification of any differences between men and women in the development of diabetes, treatment given and the management of diabetes and it complications.

Taken together, addressing these knowledge gaps will provide new insights into the mechanisms underpinning sex differences in the association between diabetes and vascular diseases, which, in turn, will help inform policies to ensure that women are not disproportionately affected by diabetes, and will help to reduce the burden in both sexes.
